# Protein kinase C-delta inhibition protects blood-brain barrier from sepsis-induced vascular damage

**DOI:** 10.1186/s12974-018-1342-y

**Published:** 2018-11-06

**Authors:** Yuan Tang, Fariborz Soroush, Shuang Sun, Elisabetta Liverani, Jordan C. Langston, Qingliang Yang, Laurie E. Kilpatrick, Mohammad F. Kiani

**Affiliations:** 1 Department of Mechanical Engineering, College of Engineering, Temple University, Philadelphia, PA 19122 USA; 2 Center for Inflammation, Clinical and Translational Lung Research, Lewis Katz School of Medicine, Temple University, Philadelphia, PA 19140 USA; 3 Sol Sherry Thrombosis Research Center, Lewis Katz School of Medicine, Temple University, Philadelphia, PA 19140 USA; 4 Department of Radiation Oncology, Lewis Katz School of Medicine, Temple University, Philadelphia, PA 19140 USA

**Keywords:** Blood-brain barrier, Protein kinase C-delta, Microvascular endothelial cells, Microfluidic assay, Sepsis, Neuroinflammation

## Abstract

**Background:**

Neuroinflammation often develops in sepsis leading to activation of cerebral endothelium, increased permeability of the blood-brain barrier (BBB), and neutrophil infiltration. We have identified protein kinase C-delta (PKCδ) as a critical regulator of the inflammatory response and demonstrated that pharmacologic inhibition of PKCδ by a peptide inhibitor (PKCδ-*i*) protected endothelial cells, decreased sepsis-mediated neutrophil influx into the lung, and prevented tissue damage. The objective of this study was to elucidate the regulation and relative contribution of PKCδ in the control of individual steps in neuroinflammation during sepsis.

**Methods:**

The role of PKCδ in mediating human brain microvascular endothelial (HBMVEC) permeability, junctional protein expression, and leukocyte adhesion and migration was investigated in vitro using our novel BBB on-a-chip (B^3^C) microfluidic assay and in vivo in a rat model of sepsis induced by cecal ligation and puncture (CLP). HBMVEC were cultured under flow in the vascular channels of B^3^C. Confocal imaging and staining were used to confirm tight junction and lumen formation. Confluent HBMVEC were pretreated with TNF-α (10 U/ml) for 4 h in the absence or presence of PKCδ-*i* (5 μM) to quantify neutrophil adhesion and migration in the B^3^C. Permeability was measured using a 40-kDa fluorescent dextran in vitro and Evans blue dye in vivo.

**Results:**

During sepsis, PKCδ is activated in the rat brain resulting in membrane translocation, a step that is attenuated by treatment with PKCδ-*i*. Similarly, TNF-α-mediated activation of PKCδ and its translocation in HBMVEC are attenuated by PKCδ-*i* in vitro. PKCδ inhibition significantly reduced TNF-α-mediated hyperpermeability and TEER decrease in vitro in activated HBMVEC and rat brain in vivo 24 h after CLP induced sepsis. TNF-α-treated HBMVEC showed interrupted tight junction expression, whereas continuous expression of tight junction protein was observed in non-treated or PKCδ-*i*-treated cells. PKCδ inhibition also reduced TNF-α-mediated neutrophil adhesion and migration across HBMVEC in B^3^C. Interestingly, while PKCδ inhibition decreased the number of adherent neutrophils to baseline (no-treatment group), it significantly reduced the number of migrated neutrophils below the baseline, suggesting a critical role of PKCδ in regulating neutrophil transmigration.

**Conclusions:**

The BBB on-a-chip (B^3^C) in vitro assay is suitable for the study of BBB function as well as screening of novel therapeutics in real-time. PKCδ activation is a key signaling event that alters the structural and functional integrity of BBB leading to vascular damage and inflammation-induced tissue damage. PKCδ-TAT peptide inhibitor has therapeutic potential for the prevention or reduction of cerebrovascular injury in sepsis-induced vascular damage.

## Background

Sepsis is a life-threatening organ dysfunction caused by a dysregulated host response to infection [1]. It is one of the leading causes of death in ICUs causing more than 200,000 deaths/year in the USA [2, 3]. Patients who recover from sepsis suffer from impaired quality of life and rapid degradation in cognition and functional capacity which is more pronounced in middle-aged and older survivors [4].

During sepsis, the endothelium is an active participant in the recruitment and activation of neutrophils through the production of chemokines/cytokines and expression of adhesion molecules [5–7]. Sepsis induces activation of cerebral endothelial cell (EC) which initiates a cascade of proinflammatory events by releasing various mediators into the brain [8], resulting in alterations in the blood-brain barrier (BBB), leukocyte dysregulation, and subsequent brain tissue damage [9]. A key step in neutrophil-mediated brain damage is the migration of neutrophils across the damaged BBB. BBB properties are primarily determined by tight and adherens junctions between the cerebral EC [10]. Normally, junctional complexes prevent the transmigration of blood cells. However, in sepsis, BBB disruption leads to the influx of neutrophils into brain tissue. To date, there are no specific pharmacological therapies available that protect brain from neutrophil-mediated tissue damage [2, 11].

Our group has identified protein kinase C-delta (PKCδ) as a critical regulator of the inflammatory response and an important regulator of endothelial proinflammatory signaling [12–18]. PKCδ inhibition had an anti-inflammatory and lung protective effect indicating that targeting PKCδ may offer a unique therapeutic strategy for the protection of EC and control of neutrophil-induced tissue damage [16, 18]. PKCδ is a member of the protein kinase C (PKC) superfamily. While PKCδ has been identified as an important regulator of inflammation, the mechanisms by which PKCδ regulates BBB permeability, EC adhesion molecule/junctional protein expression, and neutrophil migration in sepsis are incompletely understood and further studies are needed to elucidate the regulation and relative contribution of PKCδ in the control of individual steps in this process.

Given the complexity of existing in vivo models of the inflammatory process, several in vitro models have been developed. While 2D flow chambers can be used to examine adhesion molecule/junctional protein expression, as well as neutrophil rolling/adhesion phenomena, they lack the appropriate geometry to model EC permeability/TEER changes and neutrophil transmigration. Boyden/transwell chambers can be used for migration studies, however do not account for in vivo fluid shear and size/topology of microvessels which is essential for the expression of junctional proteins or provide real-time visualization of the above-mentioned events. As there are no models that can monitor all these critical parameters and events in a single assay, the understanding of the inflammation cascade and the development of anti-inflammatory drugs has been hindered. In this study, we have modified our previously developed novel blood-brain barrier on-a-chip (B^3^C) microfluidic assay [19] so that it resolves and facilitates real-time assessment of the characteristics of the BBB as well as individual steps including rolling, firm arrest, spreading, and migration of neutrophils into the extra-vascular tissue space in a single system. This integrated microfluidic assay was then used to study the role of PKCδ in the modulation of each individual steps involved in inflammation of the brain during sepsis in a realistic microvasculature geometry with physiological shear conditions which allows direct observation and quantification of permeability, protein expression, leukocytes rolling, adhesion, and migration over time.

The objective of this study is to test the hypothesis that inhibition of PKCδ prevents activation of EC, protects BBB structural integrity, prevents neutrophil migration, and attenuates the development of brain inflammation. This study will provide important insight into the molecular mechanisms and functional role of PKCδ in the underlying pathophysiology of brain inflammation during sepsis and will ascertain whether targeting PKCδ offers a unique therapeutic strategy for the control of BBB damage in sepsis.

## Materials and methods

### Materials, equipment, and reagents

A rabbit polyclonal anti-rat PKCδ (Ser643/676) antibody was purchased from Cell Signaling Technology (Beverly, MA). A rabbit polyclonal anti-human TJP1/Tight Junction Protein 1 antibody was purchased from Boster Biological Technology (Pleasanton, CA); Alexa Fluor® 568 goat anti-rabbit polyclonal antibody and Alexa Fluor® 488 Phalloindin were purchased from Life Technologies Corporation (Carlsbad, CA). Human fibronectin was obtained from BD Biosciences (San Jose, CA). Human brain microvascular endothelial cell (HBMVEC), human astrocytes, endothelial cell media (ECM), and astrocyte media were purchased from ScienCell (Carlsbad, CA). Subcellular Protein Fractionation Kit, bovine serum albumin (BSA), phosphate buffered saline (PBS), Hanks’ Balanced Salt solution (HBSS), Trypsin/EDTA, formalin, Triton X-100, Draq5, 40 kDa Texas Red-conjugated dextran, and Hoechst 33342 were purchased from Thermofisher Scientific (Rockford, IL). Formamide was purchased from MilliporeSigma (Burlington, MA). B^3^C microfluidic assay platform was manufactured at the Synvivo, Inc. (Huntsville, AL).

A Nikon TE200 fluorescence microscope equipped with an automated stage was used for performing experiments. Images were acquired using an ORCA Flash 4 camera (Hamamatsu Corp., USA). An Olympus FluoView FV1000 confocal microscope equipped with a fully automated stage was used for capturing confocal image stacks. PhD Ultra Syringe pump (Harvard Apparatus, USA) was used for injecting growth media, permeability dye, or neutrophil/microparticle suspension to the B^3^C with high precision. A stage warmer was used to keep the B^3^C at 37 °C. NIS Elements software (Nikon Instruments Inc., Melville, NY) was used to control the microscope stage and the camera.

### Synthesis of PKCδ-TAT inhibitor peptide

A peptide antagonist (PKCδ-TAT) was synthesized to selectively inhibit PKCδ activity. The peptide, derived from the first unique region (V1) of PKCδ (SFNSYELGSL: amino acids 8–17), was coupled to a membrane-permeant peptide sequence in the HIV TAT gene product (YGRKKRRQRRR: amino acids 47–57 of TAT) via an N-terminal Cys-Cys bond [20]. The resulting PKCδ-TAT peptide produces a unique dominant-negative phenotype that effectively inhibits activation of PKCδ but not other PKC isotypes. The PKCδ-TAT inhibitory peptide was synthesized by Mimotopes (Melbourne, Australia) and purified to > 95% by HPLC.

### In vivo sepsis model

Animal procedures and handling were conducted in accordance with the NIH standards and were approved by the Institutional Animal Care and Use Committee at Temple University. Male Sprague-Dawley rats (300–350 g) (Charles River, Boston, MA) were used in all experiments. Rats were acclimated for at least 1 week in a climate-controlled facility and given free access to food and water. Sepsis was induced by the cecal ligation and puncture (CLP) method as described previously [21, 22]. Briefly, a midline laparotomy was performed and the cecum identified, the mesentery trimmed, and the stalk joining the cecum to the large intestine was ligated. The cecum was punctured with a 21-gauge needle, stool expressed and the cecum returned to the abdomen, and the incision closed in two layers. Sham controls underwent a laparotomy without cecal ligation or puncture. Following CLP or sham surgery, the abdominal incision was closed, and the animals were orally intubated with a 16-gauge intravenous cannula and randomized to receive either the PKCδ-TAT inhibitory peptide (200 μg/kg in 200 μl of PBS) or a like volume of PBS (vehicle).

### PKCδ phosphorylation and translocation in rats

At 24 h post-surgery, animals were euthanized and the brains were harvested. Cell membrane and cytoplasm fractions of brain tissue were isolated using a Subcellular Protein Fractionation Kit for Tissues. For Western blot analysis, isolated tissue samples were mixed with 2X sample buffer to a final concentration of 30 μg/lane and heated for 5 min at 95 °C. Purity of membrane and cytosolic fractions were routinely monitored by probing cell membrane marker VE-cadherin. Proteins were separated on 4–12% SDS-PAGE gels and transferred to nitrocellulose membranes for blotting. The presence of phosphorylated PKCδ in membrane and cytoplasm fractions was determined by a phospho-specific PKCδ (Ser643/676) antibody [23–25]. PKCδ membrane translocation was then quantified by densitometry analysis to Western blot films in ImageJ software, and the values were expressed as a ratio of membrane fraction density to cytosolic fraction density.

### Permeability measurements in vivo

Twenty-four hours post-sham or CLP surgery, animals were anesthetized, and Evans blue dye (4% in saline) was given at 2 ml/kg via tail vein. Thirty minutes post-dye injection, each rat was perfused with 50 ml of saline by direct injection through left ventricle into the ascending aorta. Brain samples were then collected, weighed, and homogenized in PBS. Evans blue was extracted from tissue homogenates by incubating samples in formamide at 60 °C for 14–18 h. The concentration of Evans blue in brain homogenate supernatants was quantified by a dual wavelength spectrophotometric method at absorptions of 620 and 740 nm that allows for correction of contaminating heme pigments using the following formula:

1 $$ E620\left(\mathrm{corrected}\right)=E620-\left(1.426\times E740+0.030\right) $$

Data are expressed as micrograms per milligram brain weight.

### Design and fabrication of the B^3^C microfluidic assay

**Fig. 1:**
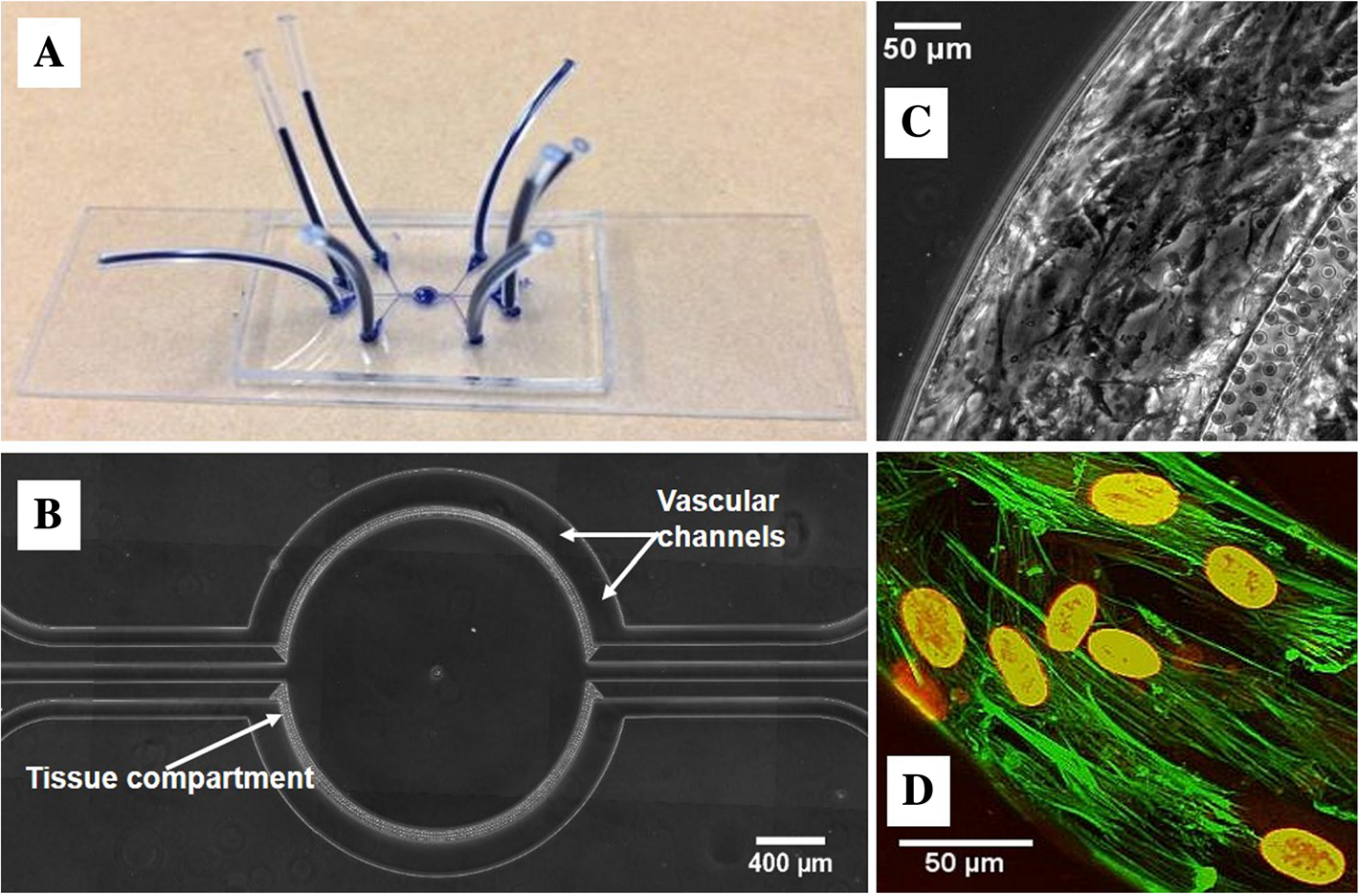
HBMVEC cultured under flow in the vascular channel of B^3^C form a complete lumen. The B^3^C is assembled on a microscope glass slide (**a**) with plastic tubes (dark blue) allowing access to individual vascular channels and the tissue compartment (**b**). Magnified (**c**) view shows HBMVEC were cultured to confluence in the vascular channels. 3D reconstruction of confocal images (**d**) of HBMVEC stained with f-actin (green) and Draq5 (red) after 72 h of flow culture (0.1 μl/min)

The blood-brain barrier on a chip (B^3^C) microfluidic assay used in this study (Fig. 1) is based on a modification of our previous design [19]. Vascular channels as well as tissue compartment were reproduced on a glass slide using soft-lithography processes as reported previously [19, 26]. This B^3^C microfluidic assay consists of vascular channels, which were covered with human brain microvascular endothelial cells (HBMVEC), in connection with a tissue compartment via a porous barrier. Microfabricated pillars (10 μm diameter) were used to fabricate the 3 μm × 100 μm pores resulting in vascular channels connected to a tissue compartment via 3 μm porous barrier, which is the optimum size for neutrophil migration.

### Culturing of endothelial cells in B^3^C

HBMVEC and human astrocytes were cultured in their corresponding culture media and used between passages 1 and 2. The astrocyte-conditioned media (ACM) was prepared by culturing 10^7^ astrocytes in 75 cm^2^ culture flask with 12 ml of growth media for 48 h, after which the media were collected and filtered as reported previously [27]. The collected ACM was mixed with fresh ECM at 50/50 ratio and was used as the culture media for EC in B^3^C. Before EC seeding, the B^3^C was first degassed, washed with sterile deionized water, and then coated with human fibronectin at 37 °C for 30 min to facilitate cell attachment. HBMVEC suspended in ECM at a concentration of 5 × 10^6^ cells/ml were seeded into the B^3^C using a programmable syringe pump and incubated at 37 °C for 4 h prior to shear flow (0.1 μl/min at the entry of the network) for 48 h. HBMVEC in B^3^C formed a confluent lumen and aligned in the direction of flow (Fig. 1). Formation of the 3D lumen in vascular channels under physiological conditions was confirmed using confocal microscopy [19, 28]. Assays in which neutrophils freely entered the tissue compartment without attachment were discarded.

### Permeability and transendothelial electrical resistance measurements in B^3^C

HBMVEC integrity in the vascular channels was quantified by measuring the flux of a 40-kDa Texas Red fluorescent dextran (25 μM in ECM) from the vascular to the tissue compartment. The vascular channels were connected to a Hamilton gas-tight syringe filled with dextran solution maintained at 37 °C mounted on a programmable syringe pump. The B^3^C was then mounted on a Nikon TE200 fluorescence microscope equipped with a temperature controllable automated stage. Permeability was measured by imaging the B^3^C every minute for 2 h while the dextran solution flowed through the vascular channel (flow rate 0.1 μl/min). Using our previously published method [19, 27], the following equation was used to calculate permeability (P) of dextran across the endothelium in B^3^C:

2 $$ P=\frac{1}{I_{{\mathrm{v}}_0}}\frac{V}{S}\frac{d{I}_{\mathrm{t}}}{dt} $$

where *I*_*t*_ is the average intensity in the tissue compartment, ${I}_{v_0}$ is the maximum fluorescence intensity of the vascular channel, and $\frac{V}{S}$ is the ratio of vascular channel volume to its surface area.

Transendothelial electrical resistance (TEER) was measured following our established method [19] using an electrode compartment outside the vascular channels. Ag/AgCl electrodes were placed on either side of the HBMVEC in the vascular and tissue compartments and connected to SynVivo Cell Resistance Analyzer (SynVivo Inc., Huntsville, AL). Impedance measurements were acquired at 10 kHz with a voltage of 10 mV. Baseline TEER of the confluent EC monolayer was determined and then at 0, 24, and 48 h following the addition of TNF-α.

### Neutrophil adhesion and migration in B^3^C

Following informed consent, human heparinized blood was obtained from healthy male or female adult donors. Human neutrophils were isolated by ficoll-hypaque separation, dextran sedimentation, and hypotonic lysis to remove erythrocytes [21, 23]. Isolated neutrophils were suspended in HBSS (5 × 10^6^ cells/ml) and labeled using CFDA/SE probe for 10 min at room temperature. All procedures were approved by the Temple University Institutional Review Board (Philadelphia, PA, USA).

Neutrophils were introduced into the vascular channels of the B^3^C at a flow rate of 0.1 μl/min. Neutrophils in contact with EC that did not move for 30 s were considered adherent. Adhesion level of neutrophils to the endothelium reached steady state after 10 min of flow and was quantified by scanning the entire network [28]. The number of migrated neutrophils was quantified using time-lapse imaging every 3 min for 60 min.

### Immunofluorescence staining of the EC in B^3^C

To study morphological changes in cells, actin filaments were stained with phalloidin and cell nucleus was stained with Hoechst 33342. To examine EC barrier function after sepsis with or without PKCδ-*i* treatment, the formation of endothelial cell-to-cell tight junction was characterized using immunostaining against zonula occludens-1 (ZO-1). Briefly, the B^3^C was perfused with 4% neutral buffered formalin to fix the cells followed by 10-min treatment with 0.1% Triton X-100 to expose ZO-1 protein. After blocking with 5% goat serum in PBS for 1 h at 37 °C, the vascular channel of the B^3^C was incubated with mouse monoclonal primary antibody against ZO-1 (1:100) overnight at 4 °C. On the second day, the B^3^C was then incubated with fluorophore-conjugated secondary antibodies Alexa fluor 594 goat anti-mouse IgG for 1 h at 37 °C. Cells in B^3^C were washed with PBS containing 5% serum between each step using a syringe. Images were taken using the same microscope and camera system as described before. The background noise was removed from the image by thresholding, and the ZO-1 staining was enhanced in the ImageJ software using the “Find Edges” function.

### PKCδ phosphorylation and translocation in HBMVEC

The presence and subcellular distribution of phosphorylated PKCδ in HBMVEC was determined by immunostaining followed by fluorescence imaging. PKCδ phosphorylation was quantified by intensity analysis in ImageJ software, and the values were expressed as a ratio of cell nucleus intensity to cytosolic intensity. HBMVEC cultured in chamber slides were fixed with 4% neutral buffered formalin followed by 0.1% Triton X-100 permeabilization. After blocking with 5% goat serum in PBS for 1 h at 37 °C, HBMVEC were incubated with phospho-specific PKCδ (Ser643/676) antibody (1:100) overnight at 4 °C. On the second day, the cells were washed and then incubated with Alexa fluor 594 fluorescent goat anti-rabbit secondary antibody for 1 h at 37 °C. Cells were washed with PBS containing 5% serum between each step. Images were taken using the same microscope and camera system as described before.

### Data analysis

Nikon Elements and Fiji software were used to collect and analyze the data [29]. Data are presented as mean ± SEM. Statistical significance was determined by one-way or two-way analysis of variance (ANOVA) with Tukey-Kramer post hoc using SigmaPlot software. Differences were considered statistically significant if *p* < 0.05.

## Results

### Brain EC form a complete lumen in B^3^C

The schematic of the B^3^C microfluidic assay is shown in Fig. 1a. Two independent vascular channels with dimensions of 200 μm (width) × 100 μm (height) × 2762 μm (length) were placed around the tissue compartment. The dimensions of the vascular channels which closely approximate the size and morphology of microvessels in vivo permit the B^3^C to maintain physiologically relevant shear flow conditions for HBMVEC growth (Fig. 1c). Simultaneous real-time visualization of the vascular and tissue compartments was achieved by the side-by-side placement of optically clear polydimethylsiloxane (PDMS) onto a glass slide. The vascular channels and tissue compartment were separated by a porous interface which was constructed by a tightly packed cylindrical micro-pillar array to allow for biochemical and cellular communications. Previously, we have reported that brain EC barrier function was dependent on the presence of astrocytes or ACM [19]. Moreover, no significant differences were detected in EC permeability, TEER, or ZO-1 expression when comparing EC treated with ACM vs. EC co-cultured with astrocytes [19]. Therefore, in this study, HBMVEC was cultured with ACM in the vascular channels without astrocytes in the tissue compartment. To allow for real-time monitoring of EC barrier function as well as neutrophil-endothelial interaction, the B^3^C is constructed from optically clear PDMS assembled on a microscope slide (Fig. 1b). HBMVEC cultured with ACM under flow formed a complete 3D lumen in the vascular channels (Fig. 1d), which mimics the normal EC lining observed in vivo.

### Sepsis-induced PKCδ activation and BBB barrier damage in rats are attenuated by treatment with a PKCδ peptide inhibitor

**Fig. 2:**
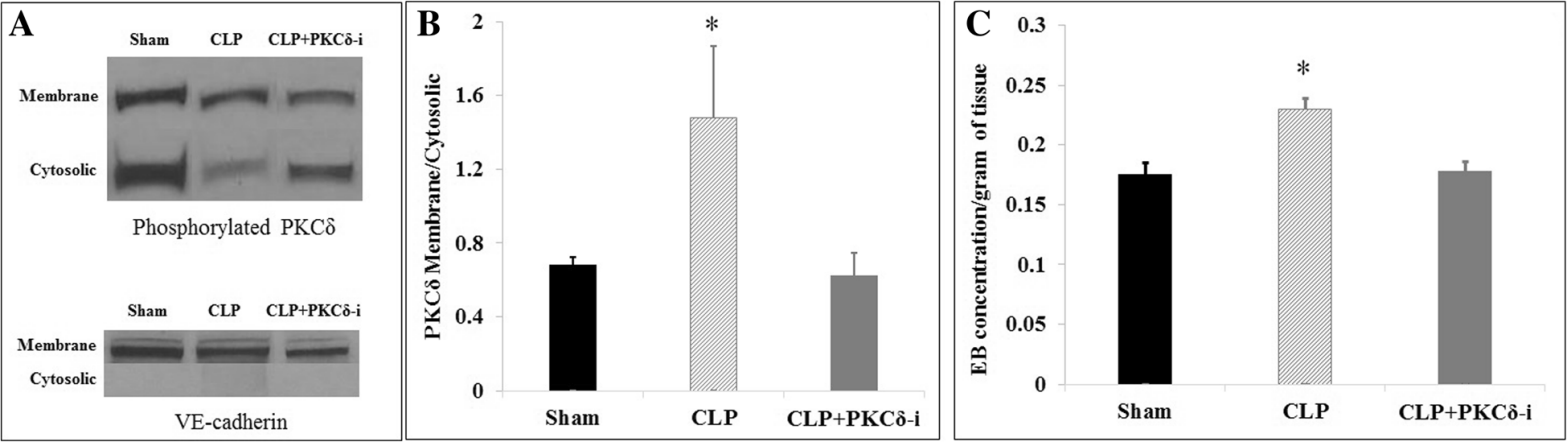
Sepsis-induced PKCδ activation and BBB barrier damage in rats are attenuated by PKCδ inhibition. **a** Representative Western blot images of phosphor-PKCδ membrane and cytosolic fractions in the brain samples of sham-operated, septic (CLP), or treated septic (CLP+PKCδ-*i*) rats. VE-cadherin was used as a marker for the membrane fraction. **b** Densitometry analysis of phosphorylated PKCδ (Ser643) translocation. Values are expressed as the density ratio of the membrane to the cytosolic fraction. **c** PKCδ inhibition (PKCδ-*i*) also attenuates sepsis (CLP) induced Evans blue (EB) dye extravasation in rat brain. Data are presented as mean ± SEM (*n* = 3). *p < 0.05 compared to sham and CLP+PKCδ-*i* by ANOVA with Tukey-Kramer post hoc

PKCδ activation requires phosphorylation on key serine/threonine sites, and translocation of PKCδ from the cell cytosol to membrane sites is a critical step in the activation of PKCδ in brain inflammation [23, 30]. To demonstrate that sepsis leads to PKCδ activation in the rat brain, we performed Western blot analysis on the subcellular fractionation of brain homogenates. As shown in Fig. 2a, in sham-operated rats, the majority of phosphorylated PKCδ was located in the cytosolic fraction as compared to the membrane fraction. In contrast, at 24 h post-CLP surgery, there was a significant increase in translocation of PKCδ from the cytosolic site to the membrane site. The PKCδ translocation pattern in CLP rats treated with the PKCδ-*i* was similar to that of sham-operated animals, indicating that the PKCδ activity was inhibited in treated animals. Densitometric analysis (Fig. 2b) of the Western blot images demonstrated that PKCδ translocation in septic rat brains was significantly increased and that treatment of septic animals with PKCδ-*i* inhibited this translocation. This pattern of PKCδ phosphorylation is consistent with our in vivo observations where PKCδ inhibition significantly reduced sepsis-induced Evans blue extravasation into the brain tissue at 24 h post-CLP surgery (Fig. 2c).

### Inflammation-mediated activation of PKCδ in HBMVEC is inhibited by treatment with a PKCδ peptide inhibitor

**Fig. 3:**
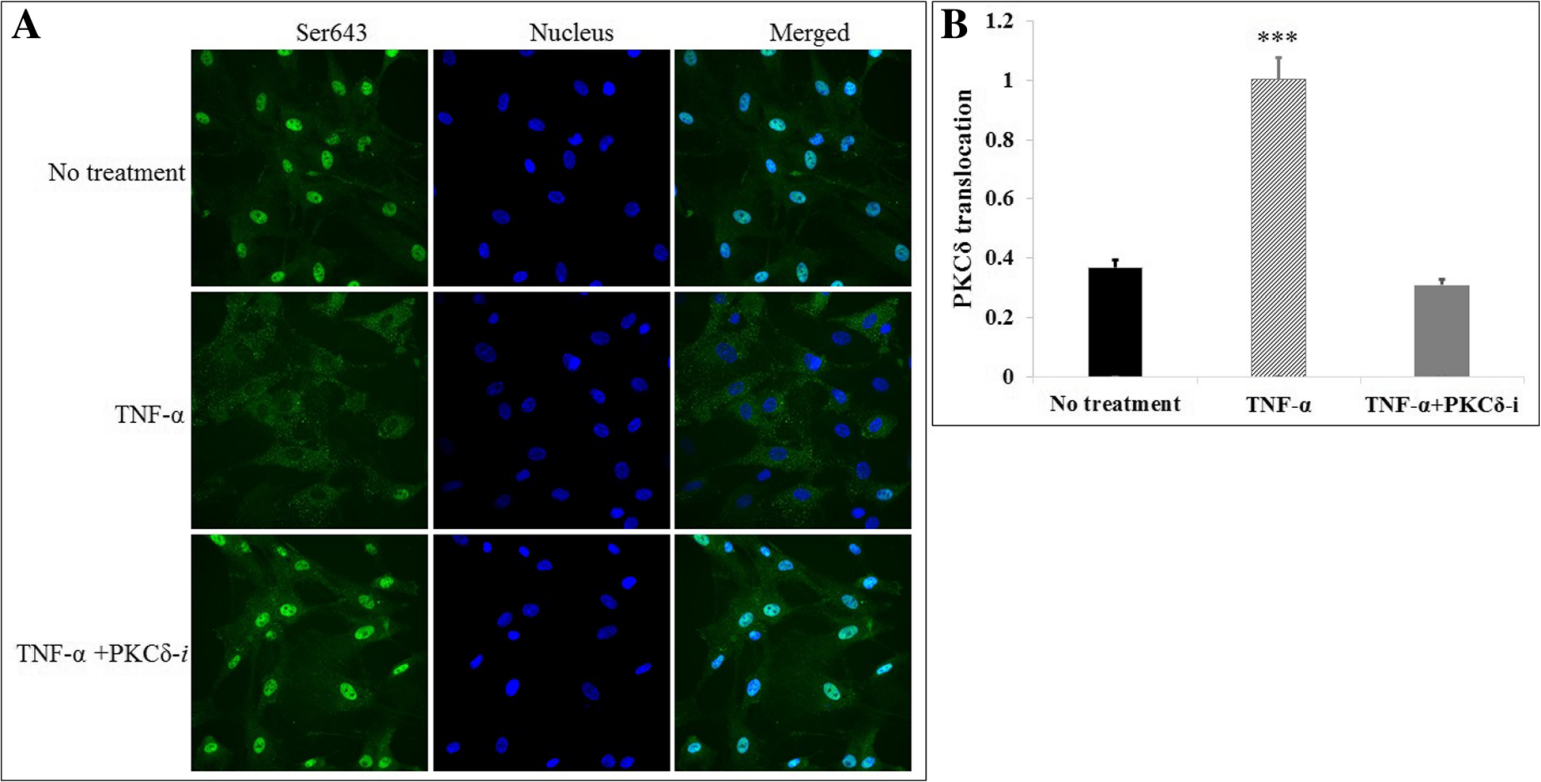
Cytokine-induced PKCδ phosphorylation in vitro is attenuated by PKCδ inhibition. **a** Representative immunostaining images of phosphor-PKCδ distribution in non-treated, TNF-α-activated, or TNF-α + PKCδ-*i*-treated HBMVEC in static culture. **b** Fluorescence intensity analysis of phosphorylated PKCδ (Ser643) translocation. Values are expressed as the intensity ratio of the cell nucleus to the cytosol. Data are presented as mean ± SEM (*n* = 3). ***p < 0.0001 compared to no treatment and TNF-α + PKCδ-*i* by ANOVA with Tukey-Kramer post hoc

To examine the effect of inflammation on PKCδ activation in brain endothelial cells, we determined PKCδ phosphorylation by immunostaining of HBMVEC in culture. As shown in Fig. 3a, in response to TNF treatment, there was a significant increase in PKCδ phosphorylation and enzyme translocation as compared to cells treated with buffer alone (no treatment). The addition of the PKCδ peptide inhibitor (TNF-α + PKCδ-*i*) attenuated TNF-mediated phosphorylation and translocation of PKCδ. This observation was confirmed by fluorescence intensity analysis as shown in Fig. 3b.

### PKCδ inhibition modulates increased permeability in activated HBMVEC in B^3^C

**Fig. 4:**
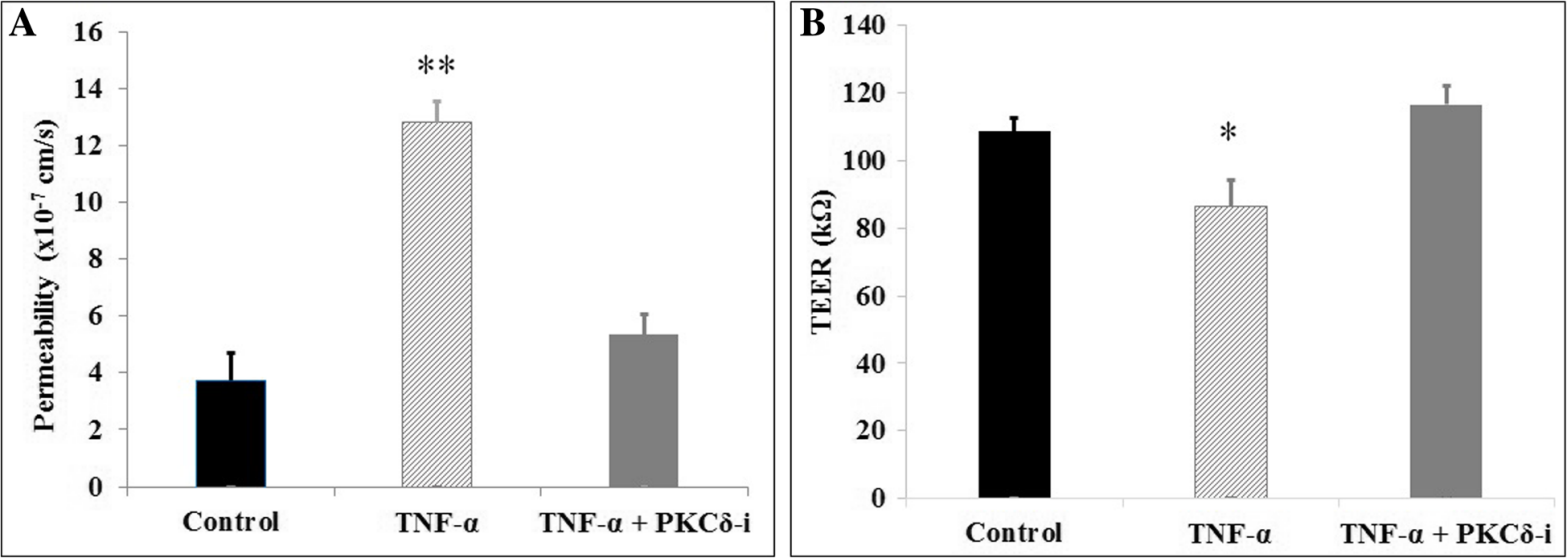
PKCδ inhibition (PKCδ-*i*) attenuates TNF-α-induced permeability increase (**a**) and TEER decrease (**b**) in vitro in B^3^C after 4 h of TNF-α activation. Data are presented as mean ± SEM (*n* = 3). **p < 0.01, *p < 0.05, compared to control and TNF-α + PCKδ-*i* treatment group by ANOVA with Tukey-Kramer post hoc

In vitro, the HBMVEC permeability was quantified in the B^3^C with no treatment or 4 h after TNF-α with or without PKCδ-*i* treatment. As shown in Fig. 4a, a threefold increase was observed in dextran permeability from vascular channels to the tissue compartment in TNF-α-treated HBMVEC (3.7 ± 1.0 × 10^−7^ to 12.8 ± 0.7 × 10^−7^ cm/s). Significant reduction in dextran permeability was observed when TNF-α-activated HBMVEC (5.3 ± 0.8 × 10^−7^ cm/s) were treated with the PKCδ-*i*.

### PKCδ inhibition modulates TEER decrease in activated HBMVEC in B^3^C

Our microfluidic system has electrodes in the two compartments for TEER measurements. Under static conditions, TEER of HBMVEC remained relatively constant over 96 h at 50 kΩ (data not shown). Under shear flow conditions, tight junctional endothelial integrity was significantly enhanced and TEER values increased by more than twofold (108.6 ± 4.0 kΩ). Addition of TNF-α produced a significant decrease in TEER indicating decreased EC barrier, whereas treatment with PKCδ-*i* of TNF-α-activated HBMVEC modulated this decrease to the control level (116.7 ± 5.4 kΩ) (Fig. 4b).

### PKCδ inhibition attenuates neutrophil adhesion and migration in B^3^C

**Fig. 5:**
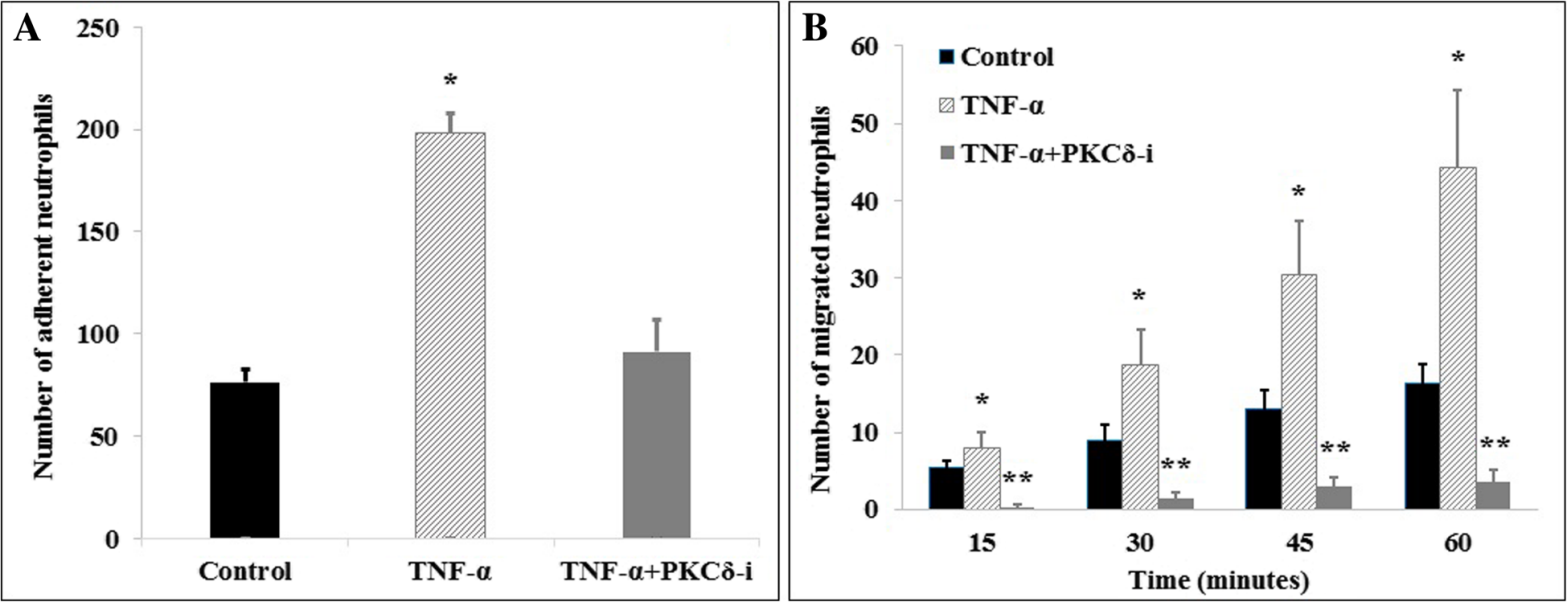
PKCδ inhibition (PKCδ-i) reduces neutrophil adhesion (**a**) and migration (**b**) in B^3^C in vitro. Data are presented as mean ± SEM (*n* = 3). **p < 0.01, *p < 0.05 compared to the other two groups by ANOVA with Tukey-Kramer post hoc

To further explore the effect of PKCδ inhibition on neutrophil-endothelial cell interaction during sepsis, neutrophil adhesion to and migration across HBMVEC under shear flow was investigated. Cytokine activation (4 h TNF-α treatment) significantly increased the number of adhering neutrophils to ECs as compared to controls (77 ± 7 vs. 199 ± 10). PKCδ inhibition significantly reduced the total number of adhered neutrophils by 54%, a level which is not statistically different from control levels (77 ± 7 vs. 91 ± 16) (Fig. 5a).

Neutrophil migration across HBMVEC into the tissue compartment was used to further assess endothelial barrier function after cytokine activation with or without PKCδ inhibition. In B^3^C, the number of migrated neutrophils across TNF-α-activated endothelium in response to the chemoattractant (fMLP) significantly increased over 60 min. Neutrophil migration across cytokine-activated HBMVEC was almost completely inhibited after PKCδ-TAT treatment (Fig. 5b), making it significantly lower than the control level of neutrophil migration.

### PKCδ inhibition attenuates cytokine-induced tight junction damage in B^3^C

**Fig. 6:**
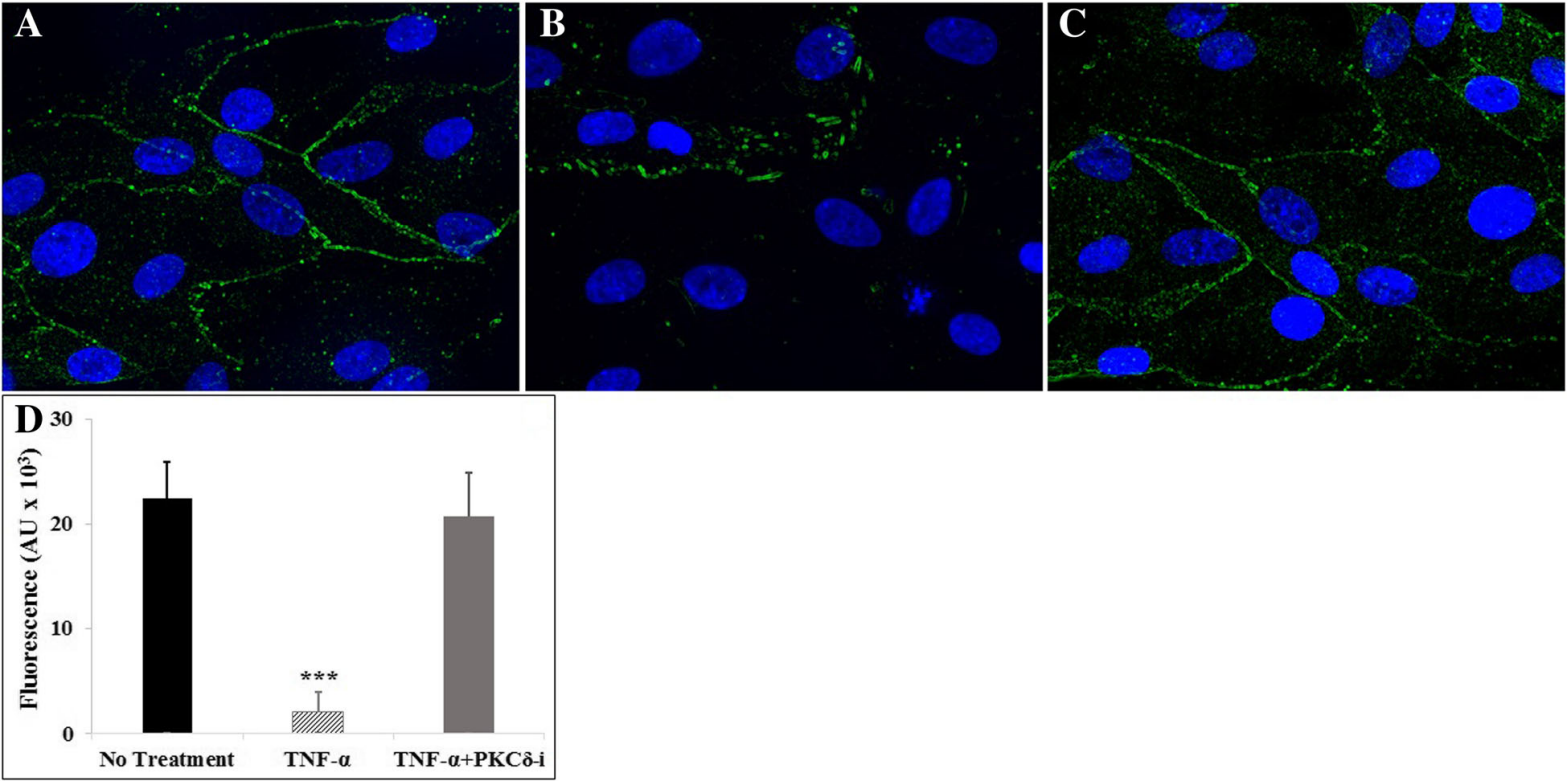
Tight junction formation by HBMVEC under flow conditions as indicated by immunofluorescence staining of ZO-1. PKCδ inhibition (PKCδ-*i*) attenuates TNF-α-induced tight junction damage in vitro in B^3^C. When cultured with normal media, tight junctions were fully established between adjacent cells (**a**). Tight junction expression was disrupted after 4 h of TNF-α activation (**b**), while PKCδ inhibition (TNF-α + PKCδ-*i*) restored tight junction expression (**c**). HBMVEC cultured for 72 h under flow (0.1 μl/min) were stained with ZO-1 (red) and Hoechst 33342 (blue). **d** Quantitative analysis to the total tight junction fluorescence intensity confirmed our observation. Data are presented as mean ± SEM (*n* = 3). *** *p* < 0.001 compared to no treatment and TNF-α + PKCδ-*i* by ANOVA with Tukey-Kramer post hoc

Tight junctions are important for regulating the barrier properties of BBB. Immunofluorescence staining for ZO-1 was used to highlight HBMVEC tight junction molecule expression under control conditions, after treatment with TNF-α, or treatment with TNF-α and PKCδ-TAT inhibitor (Fig. 6). Under control conditions, HBMVEC continuously expressed tight junctions when cultured using HA-conditioned media under shear flow in B^3^C as indicated by strong continuous ZO-1 staining in the vascular compartment (Fig. 6q). This shows that our B^3^C assay not only provides an in vivo-like shear flow environment, but also permits junction formation in ECs cultured in the vascular compartment. TNF-α activation significantly downregulated tight junction expression as indicated by a lack of ZO-1 in some cells as well as intermittent ZO-1 expression along the edges of the remaining cells (Fig. 6b). The expression of ZO-1 in PKCδ-TAT-treated, TNF-α-activated HBMVEC (Fig. 6c) was similar to those observed in control ECs (Fig. 6a), indicating that PKCδ inhibition attenuates cytokine activation-induced tight junction damage. This observation was confirmed by quantitative analysis to the total tight junction fluorescence intensity (Fig. 6d).

## Discussion

The inflammatory response is composed of multiple overlapping and redundant mechanisms, and recent research has shifted the focus to developing therapeutics that can regulate common control signaling points which are activated by diverse signals. In vitro studies using our bioinspired microfluidic assay (bMFA) demonstrated a role for pulmonary endothelial PKCδ in mediating neutrophil-endothelial interaction [28]. Studies also suggest that PKCδ may be a major mediator and/or modulator of inflammatory responses in brain [31, 32], suggesting a critical role of PKCδ in mediating BBB damage during sepsis. In this study, we demonstrate that activation of PKCδ results in alterations in tight junction protein expression and functional integrity of the BBB after cytokine activation or CLP-induced sepsis. In vitro, inhibition of PKCδ prevented activation of ECs, protected BBB structure integrity, and prevented neutrophil migration across the brain EC. Treatment of septic animals with the PKCδ inhibitor prevented activation of PKCδ and restored BBB permeability to control levels. Our findings support the hypothesis that PKCδ inhibition can attenuate the disruption of ZO-1 tight junction protein, resulting in a decrease in permeability and an increase in electrical resistance across the endothelial cell barrier.

Maintenance of normal brain function is very much dependent on the integrity of BBB that is highly selective to the passage of molecules and cells from the blood to the brain tissue. Extravasation of dyes and changes in transendothelial electrical resistance are often used to measure permeability of the BBB barrier in vivo and in vitro. For example, Evans blue is widely regarded as the standard measurement of BBB permeability and its extravasation has been used by numerous studies to quantify BBB breakdown in vivo [33–37], despite some reported limitations [38–40]. Transport of macromolecules larger than 5 nm, such as the 40-kDa dextran, occurs through the transcellular pathway (through the cell body regulated by the cell membrane lipid bilayer) [41–44] that is regulated in part by cytoskeleton proteins such as actin and is altered by TNF-α activation [45]. Under pathological conditions such as cytokine stimulations, the distribution of junctions between endothelial cells, as well as cytoskeleton proteins such as actin, can be downregulated resulting in paracellular transport of macromolecules. TNF-α stimulation has been shown to induce alterations in cell-cell and cell-matrix interaction [45]. Components of adherens (cadherin) and tight junctions (occludins) were found to be downregulated in TNF-α stimulated cells and the contacts between neighboring cells were breached [46–50]. TEER on the other hand is an index of current flow via the paracellular route (through the junctions between cells and regulated by junctional proteins) and via the transcellular route [51]. These different regulatory mechanisms may become more prominent depending on the phenomenon being studied. In this study, TNF-α activation had a larger impact on BBB permeability to a 40-kDa dextran (threefold increase) as compared to TEER (23% reduction) indicating a shift from transcellular to paracellular transport.

The role of PKC in the regulation of BBB has been studied in several disease conditions in vivo [52–55]. However, the relative contribution of each of the different PKC isoforms is still not clear. To date, there are at least 12 different PKC isoforms discovered [56]. Conflicting data on the critical role of PKCδ in these diseases have been described. For example, three PKC family isotypes (PKCα, PKCδ, and PKCε) were found to be co-localized with EC after blast exposure [52]. However, high levels of PKCθ, PKCζ, and PKCγ isozyme expression were seen in cortical endothelial cells in a rat hypoxia and post-hypoxic reoxygenation model [54]. In a rat hypertension model, sustained pharmacological inhibition of PKCδ prevented the development of hypertensive encephalopathy through prevention of BBB breakdown [55]. Utilizing an in vitro transwell co-culture model of the BBB of mouse bEnd.3 cells, Kim et al. demonstrated that both PKCβII and PKCδ were activated during aglycemic hypoxia and while PKCδ activation was found to be protective of BBB integrity, PKCβII activation was detrimental to BBB integrity [53]. Thus, whereas these studies have shown the potential role of PKC in barrier permeability, there is still some uncertainty about the specific role of the δ isoform. The differential regulation of BBB by PKCδ in diverse cell systems is not surprising as specific regulatory roles for PKCδ are context specific and dependent on mechanisms of PKCδ activation, phosphorylation patterns, and input from other signaling pathways [23, 57, 58]. To our knowledge, the present study is the first to provide direct evidence of BBB disruption caused by PKCδ activation in sepsis. Meanwhile, no studies have examined the effects of PKCδ inhibition as a therapeutic approach for treatment of sepsis-induced brain damage.

Previous studies from our group demonstrated a key role for PKCδ in the regulation of proinflammatory signaling controlling the activation and recruitment of neutrophils [14, 16, 18, 28, 59, 60]. Our recent study using a PKCδ Knock-in mouse model of sepsis and an in vitro biomimetic microfluidic assay further demonstrated that PKCδ activation (tyrosine 155 phosphorylation) is required for neutrophil activation, adherence, and transmigration through pulmonary endothelium [61]. Consistent with these findings, in the current study, we show that PKCδ not only plays a significant role in regulating EC permeability, TEER, and tight junction protein expression in activated HBMVEC in the absence of neutrophils, but also inhibits neutrophil-endothelial cell interaction.

A novel aspect of this study is assessment of vascular integrity using a biomimetic blood-brain barrier microfluidic platform (B^3^C) where EC permeability and TEER as well as neutrophil transmigration can be directly evaluated. Compared to traditional transwell-based BBB models, the permeability of our B^3^C model was significantly lower and more closely mimic reported in vivo values [19]. More importantly, B^3^C allowed for real-time monitoring of neutrophil-endothelial interaction under physiologically relevant flow conditions.

## Conclusions

We developed a first dynamic in vitro BBB on-a-chip (B^3^C) that offers the flexibility of real-time analysis and is suitable for studies of BBB function as well as screening of novel therapeutics. Our findings suggest that PKCδ activation is a key signaling event that dysregulates the structural and functional integrity of BBB, which leads to vascular damage and inflammation-induced tissue damage due to neutrophil transmigration. Our data suggest that PKCδ-TAT has therapeutic potential for the prevention or reduction of cerebrovascular injury in sepsis-induced vascular damage. Utilizing both in vivo (rat CLP model) and in vitro (B^3^C) tools, our findings support a novel therapeutic paradigm that targets PKCδ and neutrophil-endothelial interactions to protect BBB integrity and attenuate sepsis-induced brain tissue damage. These findings highlight an important control point of the proinflammatory signaling cascade and potential therapeutic targets for the treatment of sepsis-induced brain vascular damage.

## Abbreviations

ACM: Astrocyte-conditioned media
ANOVA: Analysis of variance
B^3^C: Blood-brain barrier on-a-chip
BBB: Blood-brain barrier
bMFA: Bioinspired microfluidic assay
BSA: Bovine serum albumin
CFDA/SE: Carboxyfluorescein diacetate succinimidyl ester
CLP: Cecal ligation and puncture
EC: Endothelial cell
ECM: Endothelial cell media
fMLP: N-Formylmethionyl-leucyl-phenylalanine
HBMVEC: Human brain microvascular endothelial cell
HBSS: Hanks’ Balanced Salt solution
PBS: Phosphate buffered saline
PDMS: Polydimethylsiloxane
PKC: Protein kinase C
PKCδ: Protein kinase C-delta
PKCδ-i: Protein kinase C-delta TAT peptide inhibitor
TEER: Transendothelial electrical resistance
TNF-α: Tumor necrosis factor α
ZO-1: Zonula occludens-1
